# Poster Session II - A285 DIFFERENCES IN ILEAL POUCH ANAL ANASTOMOSIS-RELATED FISTULAS VS. PERIANAL CROHN’S DISEASE

**DOI:** 10.1093/jcag/gwaf042.285

**Published:** 2026-02-13

**Authors:** R Kandel, J McCurdy

**Affiliations:** Gastroenterology, University of Ottawa, Ottawa, ON, Canada; Gastroenterology, University of Ottawa, Ottawa, ON, Canada

## Abstract

**Background:**

Perianal fistulas are a common manifestation of Crohn’s disease (CD) and occur in ∼10% of patients with ileal pouch-anal anastomosis (IPAA). It is unclear whether these fistulas share the same pathophysiology and respond similarly to medical therapy

**Aims:**

To compare the anatomy and complexity of IPAA-related fistulas (IPAA-RF) with perianal Crohn’s disease (PFCD) fistulas

**Methods:**

A retrospective comparative cohort study was conducted at The Ottawa Hospital (01/01/2004–01/01/2025). Adults (>17 years) with IPAA were matched 1:1 to PFCD patients by age and sex. Perianal, rectovaginal and rectogenitourinary fistulas were included; luminal, pouch-body fistulas and fistulas within six months of ileostomy takedown were excluded. Patients were identified by chart review using ICD-10 codes (Crohn’s, Ulcerative colitis). Fistula anatomy was classified by Park’s (anal sphincter) and complexity defined per AGA criteria.

**Results:**

We identified 66 IPAA patients and 66 matched PFCD controls. Age at diagnosis was similar (IPAA 38±11vs.PFCD 37±12 years). Smoking was more common in PFCD(40%vs.12%). PFCD patients were more likely to have multi-organ fistulas (p = 0.0006), whereas rectovaginal fistulas were more frequent in IPAA (38%vs.24%,p=0.009). There were no significant differences in anal sphincter involvement per Park’s classification. Overall rates of complex fistulas were comparable (78%vs.68%), but branching tracts were more frequent in PFCD(31%vs.12%).

**Conclusions:**

In this single-center cohort, IPAA-RF differed from PFCD, being more often rectovaginal, while PFCD showed greater multi-organ involvement and branching tracts. Although overall complexity was similar, these anatomic differences may influence management. Further studies are warranted to assess long-term outcomes in IPAA-RF.

A285 Table 1: Fistula Characteristics

**Funding Agencies:**

None

**Table:** 

	**IPAA(n)**	**%**	**PFCD(n)**	**%**	**p-value**
**AGA Anatomy**					
Simple	14	21%	15	22%	0.8130
Complex	45	68%	52	78%	0.6650
Unknown	7	11%	0	0%	
**Organ Involvement**					
Rectovaginal alone	25	38%	16	24%	0.0095
Rectogenitourinary alone	1	2%	9	13%	0.0044
Perianal alone	40	60%	50	75%	0.7413
Multiple organs involved	0	0%	13	19%	0.0006
**Park’s**					
Suubsphincteric	0	0%	0	0%	
Intersphincteric	18	27%	40	60%	0.0694
Transphincteric	16	24%	11	16%	0.0611
Suprasphincteric	3	5%	2	3%	0.6890
Extrasphincteric	0	0%	0	0%	
Unknown	25	38%	6	9%	
**Fistula tracts (mean±SD)**	1(±1.5)		2(±1.4)		0.19
**Fistula with branching**	8	12%	21	31%	0.0118
**Abscess**	26	39%	25	37%	1.0000
<2cm	8	31%	9	36%	0.9210
>2cm	18	69%	16	64%	

